# Brazing of Thin-Walled Stainless Steel Using Environmentally Friendly Ni-Cr-P Electrodeposition: Degradation Mechanism of Brazed Joint and Corresponding Improvement Strategy

**DOI:** 10.3390/ma18102406

**Published:** 2025-05-21

**Authors:** Shubin Liu, Yuqi Luan, Ikuo Shohji

**Affiliations:** 1 Provincial Key Lab of Advanced Welding Technology, School of Materials Science and Engineering, Jiangsu University of Science and Technology, 666 Changhui Road, Zhenjiang 212100, China; 18641305450@163.com; 2 Graduate School of Science and Technology, Gunma University, 1-5-1, Tenjin-cho, Kiryu 376-8515, Japan; shohji@gunma-u.ac.jp

**Keywords:** brazing characteristics, electrodeposited Ni-Cr-P interlayer, microstructure, strength property, fracture mode

## Abstract

A new brazing process for thin-walled stainless steel was proposed by combining green and efficient Ni-Cr-P electrodeposition with brazing technology. Novel information was attained by analyzing the electrodeposited Ni-Cr-P interlayers and the brazed joints and characterizing them using a combination of advanced techniques. The incorporation mechanisms of impurities (i.e., oxygen and carbon) in the Ni-Cr-P interlayers electrodeposited from a Cr(III)–glycine solution were revealed. The oxygen mainly came from the Cr(III)–hydroxy complexes formed by the hydrolysis and olation between Cr(III) complexes and OH^−^ ions near the cathode. Glycine did not directly participate in the cathode reactions but decomposed on the anode surface. These byproducts (carbonyl compounds) were directly incorporated into the interlayers in a molecular pattern, forming a weak link to the metallic chromium. Brazing test results showed that a certain amount of Cr_2_O_3_ powder, formed by the decomposition of chromium hydroxides in the interlayers under high-temperature catalysis, would cause the degradation of the brazed joints. Using the step-wise brazing method, the brazing sheets were first annealed to eliminate the impurities by utilizing the strong reducing effect of hydrogen and the weak link characteristics between carbonyl compounds and metallic chromium atoms. An excellent joint with a shear strength of 63.0 MPa was obtained by subsequent brazing. The microstructural analysis showed that the brazed seam was mainly composed of a Ni-Fe-Cr solid solution, the Ni_3_P eutectic phase, and small quantities of the Ni_5_P_2_ phase scattered in the Ni_3_P eutectic phase. Fracture mode observations showed that the cracks extended along the interface between the brittle P-containing phase and the primary phase, resulting in fracture.

## 1. Introduction

As key components of cooling circulation systems, compact heat exchangers are widely used in applications such as ships, automobiles, and solid fuel cells. With the development of high-efficiency compact heat exchangers, the stainless-steel components have become thinner and more multi-layered. To join such stainless steel, considering the need for heat resistance and corrosion resistance, Ni-based filler metals are commonly used owing to their good corrosion resistance and oxidation resistance.

Ni-Cr-P filler metal exhibits good wettability and can form ductile solid solutions in a joint with a narrow gap [1,2,3]. Moreover, compared with other melting point depressants such as silicon and boron, phosphorus is less likely to form intermetallic compounds (IMCs) and dissolve at the interface of the base metal, making it more suitable for connecting thin-walled parts. However, due to the high hardness and brittleness of Ni-based filler metals, it is difficult to supply a sufficient amount of filler metal to the brazing area, which in turn reduces the reliability of the brazed joint [4].

Coating technology is mainly used in surface anti-corrosion, wear, fatigue, etc. There are various techniques for the deposition of coatings, including electrophoretic deposition (EPD) [5], spray deposition [6], sol–gel deposition [7], and electrodeposition [8]. EPD uses electric current to attract suspended particles in a dielectric bath and deposit them on the substrate surface. However, this method has the problem of easy agglomeration of particles (especially metal particles), resulting in poor flatness of the deposited film. Spray deposition has high requirements on the production environment (such as a vacuum); furthermore, the deposition efficiency is low. Sol–gel deposition is mainly used to prepare metal-oxide/-hydroxide coatings. A high O content in the coating would damage the reliability of the brazed joint. Consequently, the properties or mechanisms of these deposition methods make them unsuitable for the preparation of the brazing filler metal. Compared with the above deposition methods, electrodeposition has been widely applied in various industrial sectors since the end of the 19th century, due to its cost-effectiveness, scalability, and versatility [8,9]. In addition, it is one of the most promising techniques for the preparation of alloy coatings of high quality because it allows a high degree of control over both the chemical composition and the thickness of the electrodeposited alloys.

Some researchers have explored the use of plated Ni-based alloy interlayers for brazing stainless steel. King et al. [10] pioneered the use of plated Ni-based coatings as a brazing filler metal. However, plated Ni-based alloy coatings are formed using an electroless nickel (EN) plating process, which has many disadvantages and is costly. Moreover, since the deposition potential of chromium is relatively negative (−1.29 V versus Ag/AgCl), metallic chromium cannot be obtained by the EN method [11]. Maeda [12] introduced a step-by-step plating method to prepare brazing interlayers. First, a 35 μm thick Ni-P alloy layer was electrodeposited on the surface of the component, and subsequently, a 15 μm thick Cr layer was deposited on the Ni-P alloy layer. The double metal layer was heat-treated to form a Ni-Cr-P alloy interlayer, thereby achieving the brazing connection of multi-layer superimposed stainless-steel plates. However, the process is complicated and reduces the efficiency of brazing. Additionally, the Cr layer is prepared by electroplating hexavalent chromium (Cr(VI)), which has been prohibited because of its significant environmental toxicity and human carcinogenicity.

Trivalent chromium (Cr(III)) plating electrolytes are a “green” and effective alternative to Cr(VI) plating solutions [13,14,15]. However, metallic chromium cannot be electrodeposited from a simple aqueous solution of trivalent chromium, but rather, a metallic chromium coating can be obtained by adding a certain amount of a complexing agent [16,17,18,19]. Formic acid [19,20] is the earliest and most practical complexing agent used in trivalent chromium electrodeposition. However, subsequent research revealed that the formic acid bath was unstable, and the deposition rate of trivalent chromium dropped sharply with increasing use time. The combination of urea and formic acid [21] can improve the deposition efficiency of trivalent chromium, but the O and C content in the deposited coating is too high. Similarly, although oxalic acid solutions [22] have relatively good stability, the deposited coating still has the problem of a high O and C content. Consequently, glycine [23,24] has been used in trivalent chromium deposition, as it is both a strong complexing agent and exhibits pH buffering properties, allowing the olation reaction to be avoided and reducing the incorporation of impurities (e.g., oxygen and carbon) in the deposited coatings.

In the present study, we propose a new brazing process for thin-walled stainless steel by combining green and efficient electrodeposition and brazing technology. The formation of a Ni-Cr-P alloy interlayer was analyzed by adjusting the solution composition and plating conditions. Furthermore, the incorporation and elimination mechanisms of impurities (i.e., oxygen and carbon) were revealed. Additionally, brazing tests were conducted to verify the brazing performance of the electrodeposited Ni-Cr-P interlayers. Finally, the microstructural and strength properties of the brazed joints were investigated. This research provides a new technical approach for achieving the high-quality and high-efficiency brazing of complex thin-walled parts.

## 2. Materials and Methods

**Figure 1:**
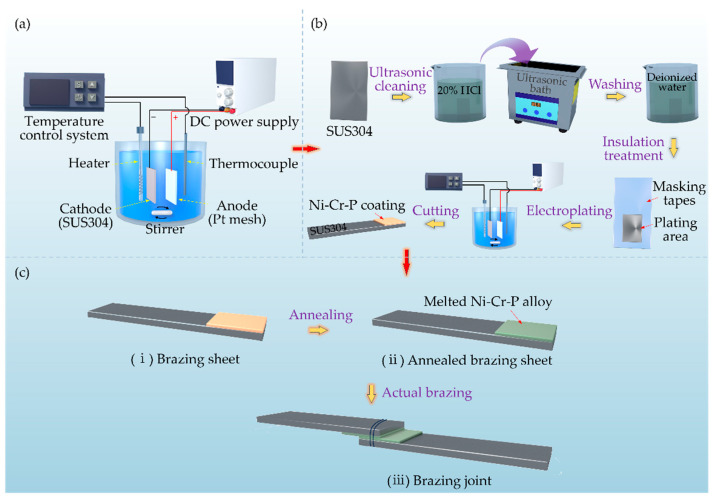
Illustration of the process of electrochemical deposition of a Ni-Cr-P interlayer and the brazing of stainless steel with the deposit as a filler metal: (**a**) electrodeposition system, (**b**) preparation of brazing sheet, and (**c**) brazing process.

Figure 1 shows the process for the electrochemical deposition of a Ni-Cr-P alloy interlayer and the brazing of stainless steel with the deposit as a filler metal. An electrochemical deposition system was established to synthesize the Ni-Cr-P alloy interlayer for a fixed-domain controllable supply of brazing filler. Therein, the temperature control system (YAMAMOTO-MS, YAMAMOTO, Tokyo, Japan) consisted of a heater and a thermocouple to control the plating temperature. A DC power supply (UTP1306S, UNI-T, Dongguan, China) was used to control the current density in the plating area. Subsequently, brazing tests were carried out on stainless steel coated with an alloy interlayer to study the brazability of the deposit, the interfacial reactions and elemental diffusion behavior between the base metal and the deposit, and the mechanical properties of the brazed joint.

### 2.1. Electrodeposition of Ni-Cr-P Alloy Interlayers

The chemical compositions of the electrolytes used for the electrochemical synthesis of the Ni-Cr-P alloy interlayers are given in Table 1. The electrolytes were prepared using analytical-grade chemicals and deionized water. Cr(III) ions, Ni(II) ions, and H_2_PO_2_^−^ ions were used as the source of the Ni-Cr-P alloy, glycine was used as a complexing agent for Cr(III), NH_4_Cl and NaCl were used as conducting salts, and NaBr was used to inhibit the generation of Cr(VI) ions at the anode [11]. In addition, sodium citrate was used in combination with boric acid to enhance the pH buffering capacity of the electrolytes, and saccharin sodium and sodium dodecyl sulfate (SDS) were used as surfactants to improve the performance of the electrodeposited alloy interlayers. The solution pH was adjusted to 1.8 by adding a 10% NaOH or HCl solution. Four conditions, referred to as S1–S4, were used to perform the electrodeposition, as shown in Table 1. Accordingly, the chemical compositions of the electrodeposited Ni-Cr-P interlayers were analyzed by X-ray fluorescence (XRF, Shimadzu XRF-1700, Shimadzu, Kyoto, Japan). To avoid interference from Cr and Ni in the SUS304 stainless steel, Cu sheets (code name C1220P, with a purity of 99.9%) with thicknesses of 1 mm were used as substrates to conduct the electrodeposition of the Ni-Cr-P alloy for chemical composition analysis.

### 2.2. Identification of Electroactive Cr(III) Complexes

Electroactive Cr(III) complexes were characterized by an ultraviolet–visible (UV-vis) spectrophotometer (U-3000, Hitachi, Tokyo, Japan). The complexing properties of glycine molecules with Cr(III) ions were analyzed by adjusting the composition of the electrolytes. The test solutions used for performing UV-vis spectrometry are shown in Table 2.

### 2.3. Materials and Brazing Tests

SUS304 stainless steel has been commonly used as a base material for heat exchangers, owing to its excellent corrosion and heat resistance. Therefore, in this study, a thin-walled SUS304 stainless-steel sheet with a thickness of 1.0 mm was used as the base metal, and a Ni-Cr-P interlayer with a mean thickness of 20 μm was used as the brazing filler metal. The chemical compositions of the stainless steel and filler metal are given in Table 3. It should be noted that a P content of 10–12 mass% in the Ni-P alloy system had the lowest melting point (approximately 890 °C) [25].

**Figure 2:**
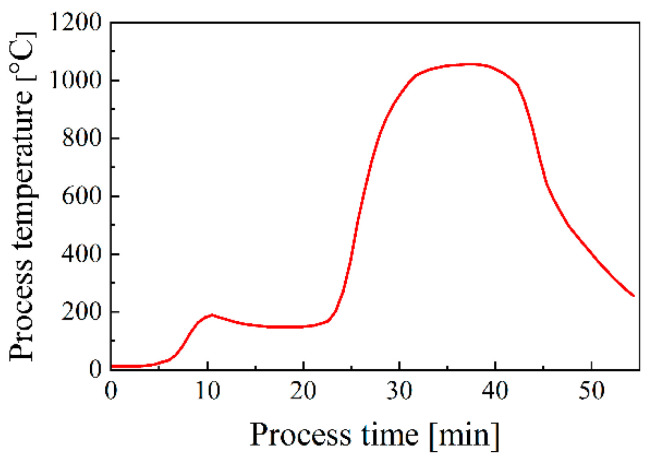
Temperature profile for brazing.

The brazing tests were performed using a hydrogen reduction furnace with a dew point of −50 °C. The temperature profile in the brazing test was recorded using a thermocouple, as shown in Figure 2. The heating time from room temperature to brazing temperature (1020 °C) and the cooling time from the brazing temperature to room temperature were approximately 30 and 45 min, respectively. The holding time at the brazing temperature was 10 min. In addition, the cooling time from 800 °C to 600 °C was controlled to less than 4 min to prevent the sensitization of SUS304. The flow quantity of hydrogen gas was 7.5 m^3^/h. In this study, a new brazing method, the “step-wise brazing method”, was introduced to conduct the brazing tests. In this approach, first, the brazing sheets (i.e., steel sheets with a Ni-Cr-P interlayer electrodeposited on one side surface; see Preparation of brazing sheet in Figure 1) were preheated via the brazing temperature. Subsequently, the preheated sheets were assembled with a thin steel wire to perform brazing. The purpose of this preheating process was to remove oxygen and carbon from the coated surface. In addition, it should be emphasized that the preheating temperature only needs to be higher than the melting point of the alloy interlayer. For convenience, the same temperature as the brazing process was set in this study.

### 2.4. Microstructural Characterization

A microstructural analysis of the brazed joints was performed with an electron probe X-ray microanalyzer (EPMA; EPMA-1610, Shimadzu, Kyoto, Japan) at an accelerating voltage of 15 kV. Before microscopic analysis, the specimens for cross-sectional observation were prepared by embedding them in an epoxy resin; they were then abraded with #500–#4000 emery papers and subsequently polished with a 1.0 μm Al_2_O_3_ suspension. A green powder was distributed on the fractured surface of the joint obtained by the direct brazing method, and a phase analysis of the powder was performed by X-ray diffraction (XRD, Rigaku RINT 2200 VF, Rigaku, Tokyo, Japan) at 40 kV and 20 mA with Cu *Kα* radiation in the 2*θ* range of 20–80°.

### 2.5. Shear Strength Properties

Shear tests were performed at room temperature using a universal testing machine (5567, Instron Japan Co., Ltd., Kanagawa, Japan) with a cross-head speed of 10 mm/min. The geometry and dimensions of the specimen used for the shear test were the same as those shown in Brazing process in Figure 1. The fracture mode observation of the brazed joints was performed via scanning electron microscopy (SEM; HITACHI S-4300SE/N, Tokyo, Japan) and optical microscopy (OM; VK-X150, KEYENCE, Osaka, Japan).

## 3. Results and Discussion

### 3.1. Electrodeposition of Ni-Cr-P Alloy

#### 3.1.1. Identification of Electroactive Cr(III) Complex

**Figure 3:**
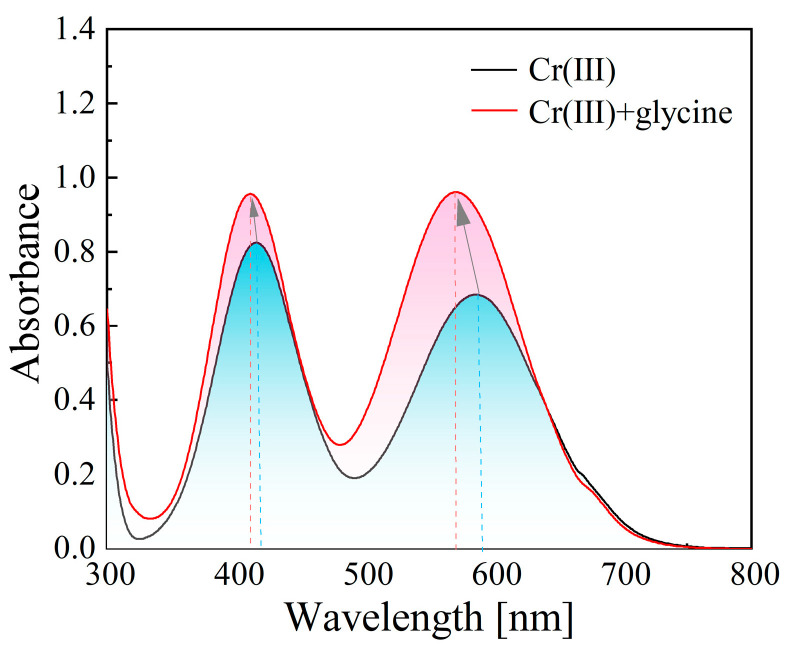
UV-vis spectrogram of trivalent chromium electrolytes with and without complexing agent (the arrows indicate the direction of peaks’ shift).

In aqueous solutions, Cr(III) ions usually exist in the form of Cr[(H_2_O)_6_]^3+^, which is an inner-orbital coordination compound according to valence bond theory. The Cr[(H_2_O)_6_]^3+^ complex exhibits strong inertness due to its regular octahedral structure, which makes it difficult for Cr(III) ions to contact the cathode to receive electrons for electroreduction [26,27]. Therefore, in order to achieve the efficient electrodeposition of metallic chromium, one of the key issues is to generate a sufficient quantity of active Cr(III) complexes in the electrolyte. Zeng et al. [28] studied the role of complexing ligands in trivalent chromium electrodeposition. They reported that the introduction of a complexing ligand would expand the distance between Cr and H_2_O, thereby accelerating the reduction of Cr(III) to metal Cr. Accordingly, if one or more H_2_O ligands are replaced by glycine molecules, the geometric structure of Cr[(H_2_O)_6_]^3+^ will be disordered, weakening the bonding between the central ions (Cr(III)) and ligands. Under the pull of cathodic electrons, Cr[(H_2_O)_6−x_L_x_]^3+^ (L denotes the complexing ligand) complex ions lose H_2_O molecules to expose Cr ions, which is propitious for metal ions that contact the cathode to react with cathodic electrons [17]. Consequently, Cr(III) ions can be reduced easily. Figure 3 shows the UV-vis spectrogram of the chromium electrolytes with and without a complexing agent. It was found that the spectral peak sizes increased while slightly shifting to the lower wavelengths after glycine was added. This can be attributed to a partial exchange of the H_2_O ligands with glycine molecules. Accordingly, the formation of electroactive Cr(III) complexes in the electrolyte was confirmed.

#### 3.1.2. Chemical Composition and Surface Morphology of Electrodeposited Ni-Cr-P Alloy

**Figure 4:**
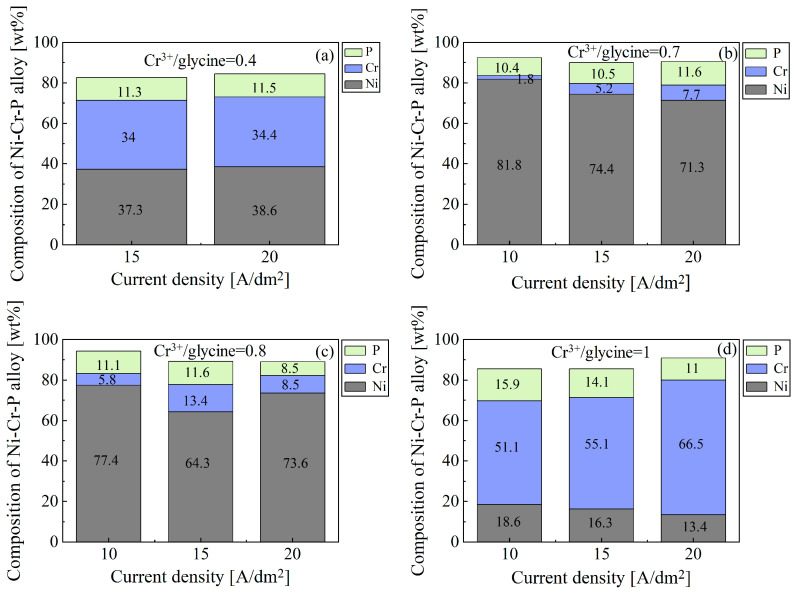
Effect of the current density on the composition of Ni-Cr-P alloys electrodeposited from electrolytes with Cr^3+^/glycine ratios of (**a**) 0.4, (**b**) 0.7, (**c**) 0.8, and (**d**) 1.

Figure 4 shows the effect of the current density on the composition of the Ni-Cr-P alloy electrodeposited from electrolytes with different Cr^3+^/glycine ratios. The Cr^3+^/glycine ratios were calculated for electrolytes S1 to S4 (Table 1). Since the formation quality of the interlayers was poor at a Cr^3+^/glycine ratio of 0.4 and a current density of 10 A/dm^2^, their composition was not measured. In addition, nickel is a matrix metal, and its content is affected by changes in the contents of other elements in the alloy, which is not discussed here. As shown in Figure 4, overall, under each Cr^3+^/glycine ratio, the Cr content increased with the increase in the current density. This is because the onset potential of Cr(III) deposition (approximately −0.98 V) is higher than that of Ni (II) (approximately −0.8 V), so a higher current density is beneficial to the electroreduction of Cr(III) [11]. In addition to current density, the concentration of electroactive Cr(III) complexes near the cathode is another important factor affecting chromium electrodeposition. When the quantity of electroactive Cr(III) complexes near the cathode was sufficient, the higher the current density was, the more metallic chromium was electroreduced. Accordingly, the Cr content decrease under a current density of 20 A/dm^2^ shown in Figure 4c can be associated with the insufficient quantity of electroactive Cr(III) complexes near the cathode. In other words, driven by higher current density, the electroactive Cr(III) complexes in the bulk solution cannot replenish the consumption of electroactive Cr(III) complexes near the cathode, thereby reducing the electroreduction of chromium. In comparison, the Cr content at Cr^3+^/glycine ratios of 0.4 and 1 was significantly higher than that at other ratios. Although the Cr content in the alloy layer is high under these conditions, it is not entirely metallic chromium, which will be discussed below. On the other hand, P did not show a similar variation pattern to that of Cr, which can be associated with the fact that the co-deposition of P occurred via a chemical reaction [11] and was not governed by the current density.

**Figure 5:**
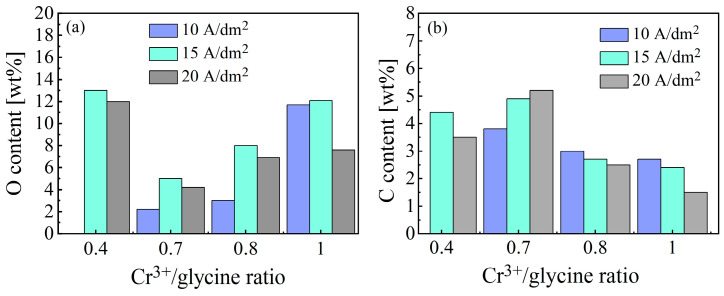
Effect of the Cr^3+^/glycine ratio on (**a**) O content and (**b**) C content in the electrodeposited Ni-Cr-P alloy interlayers.

The organic compound glycine was introduced to form active Cr(III) complexes, which improved the deposition process. However, this was a source of unwanted impurities, i.e., oxygen and carbon. Figure 5 shows the effect of the Cr^3+^/glycine ratio on the content of oxygen and carbon in the electrodeposited Ni-Cr-P alloy. As shown in Figure 5a, overall, as the Cr^3+^/glycine ratio increased from 0.4 to 1, the oxygen content decreased to a minimum at a ratio of 0.7 and then increased. However, the variation in the oxygen content in the alloy layers did not depend on the current density in a linear manner. Based on these results and the variation in the chromium content with the Cr^3+^/glycine ratio shown in Figure 4, the oxygen content in the alloy layers seems to be correlated with the chromium content. This is because the content of electroactive Cr(III) complexes formed in the electrolyte is governed by the concentration ratio of Cr^3+^/glycine, while the only species in the electrolyte that deposits metallic chromium is the [Cr(H_2_O)_4_(Gly)]^2+^ complex [23]. When the glycine concentration is low (a Cr^3+^/glycine ratio of 1), the active [Cr(H_2_O)_4_(Gly)]^2+^ complex formed in the electrolyte is insufficient, while the predominant species is the inactive Cr[(H_2_O)_6_]^3+^ complex; conversely, when the glycine concentration is high (a Cr^3+^/glycine ratio of 0.4), more glycine molecules combine with Cr(III) to form a [Cr(H_2_O)_2_(Gly)_2_]^+^ complex, which is an inactive Cr(III)–glycine species. The inactive species cannot be electroreduced at the cathode, but instead accelerates the combination of trivalent chromium ions and OH^−^ ions under the drive of the current field to form metal hydroxides (i.e., Cr-OH/-O compounds) that are co-deposited into the alloy interlayers, reducing the quality of the electrodeposited Ni-Cr-P alloy interlayers [11,23]. This also explains why the Cr and O contents in the electrodeposited alloy interlayers are relatively high at Cr^3+^/glycine ratios of 0.4 and 1 (see Figure 4a,d and Figure 5a, respectively). Moreover, the oxygen content in the alloy layers electrodeposited from the electrolyte with a Cr^3+^/glycine ratio of 1 is slightly lower than that of the alloy layers electrodeposited from the electrolyte with a Cr^3+^/glycine ratio of 0.4, which can be associated with the fact that the content of the active [Cr(H_2_O)_4_(Gly)]^2+^ complex formed in the former electrolyte is higher than that of in the latter electrolyte. Accordingly, the content of the metallic Cr in the alloy layers electrodeposited from the electrolyte with a Cr^3+^/glycine ratio of 1 is higher, thereby reducing the proportion of Cr-OH/-O compounds. Dissimilarly, the carbon content exhibited the opposite trend to that of oxygen, as shown in Figure 5b. The reason for this will be revealed in the subsequent mechanistic study of the incorporation of carbon in the electrodeposited Ni-Cr-P interlayers. Although the oxygen content was the lowest under a Cr^3+^/glycine ratio of 0.7 and a current density of 10 A/dm^2^, the Cr content in the electrodeposited Ni-Cr-P interlayer was low (approximately 1.8 wt% in Figure 4b).

**Figure 6:**
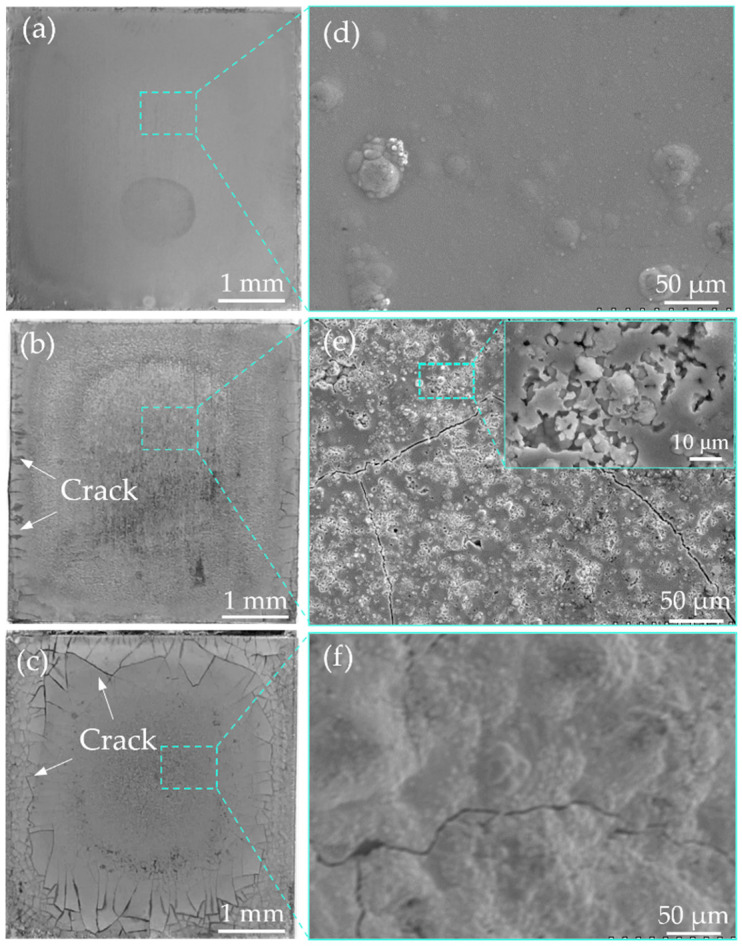
Surface morphology of the Ni-Cr-P alloy interlayers electrodeposited under different current densities from electrolytes with a Cr^3+^/glycine ratio of 0.8 ((**a**–**c**) macrographs and (**d**–**f**) micrographs (SEM)): (**a**,**d**) under a current density of 10 A/dm^2^, (**b**,**e**) under a current density of 15 A/dm^2^, and (**c**,**f**) under a current density of 20 A/dm^2^.

Figure 6 presents the morphologies of the Ni-Cr-P alloy interlayers electrodeposited under different current densities from electrolytes with a Cr^3+^/glycine ratio of 0.8. From the macrographs of the alloy interlayers (see Figure 6a–c), the interlayer obtained under a current density of 10 A/dm^2^ has a better gloss. However, as the current density increases, the glossiness of the interlayers deteriorates, and cracks appear around the interlayers and continue to extend toward the middle region. From the micrographs of the interlayers, the interlayer obtained under a current density of 10 A/dm^2^ is compact and characterized by typical modular morphology (see Figure 6d). When the current density increases to 15 A/dm^2^, the hydrogen evolution reaction on the cathode surface intensifies, forming a large number of hydrogen evolution channels in the interlayer, which causes it to become loose in texture (see Figure 6e insert). Further increasing the current density will cause “burns” around the alloy layer due to the edge effect of current distribution, making the surface uneven, as shown in Figure 6f. Combined with the above component analysis, the most suitable conditions for obtaining a Ni-Cr-P interlayer of good quality seem to be a Cr^3+^/glycine ratio of 0.8 and a current density of 10 A/dm^2^.

#### 3.1.3. O and C Incorporation Behavior During Ni-Cr-P Electrodeposition

Due to the hydrolysis and olation characteristics of Cr(III), the electrode reactions taking place during trivalent chromium electrodeposition are very complex [18,26]. Nevertheless, elemental analysis showed that no nitrogen was present in the electrodeposited Ni-Cr-P interlayers, indicating that glycine was not directly responsible for O and C incorporation. In previous work [11] on the mechanistic study of Ni-Cr-P alloy electrodeposition, the reduction of trivalent chromium to metallic chromium was confirmed to undergo a two-step process (i.e., Cr(III)→Cr(II)→Cr(0)), and the reduction potential of Cr(II)→Cr(0) was determined to be approximately −1.29 V (versus Ag/AgCl). Due to the negative overpotential for Cr(II) reduction, the hydrogen evolution, shown as follows, was severe at the cathode:

H_3_O^+^ + 2e^−^ → OH^−^ + H_2_(gas)↑. (1)

Furthermore, since the hydrogen evolution potential of H_3_O^+^ is similar to that of Cr(II) [29], a higher current density would enhance the hydrogen evolution reaction. Accordingly, more OH^−^ ions would be produced, increasing the pH near the cathode.

In addition, the temperature near the cathode increased due to the conversion of electrical energy into thermal energy. Under the combined effect of the above two factors, the hydrolysis and olation between the Cr(III) complexes and OH^−^ near the cathode was accelerated, forming Cr(III)–hydroxy complexes. As these byproducts were adsorbed on the cathode surface, glycine molecules separated from Cr(III) and returned to the bulk electrolyte [19,23]. Simultaneously, -OH groups reacted with Cr(III) to form metal hydroxides (i.e., Cr-OH/-O compounds). These compounds were mainly concentrated in the top layer of the electrodeposited Ni-Cr-P alloy, forming a dense oxide film [24,29]. The easy hydrolysis characteristics of trivalent chromium made the above cathodic side reactions occur preferentially. Consequently, this seems to have been the main pathway for O incorporation.

As the only organic compound involved in the electrodeposition process, glycine was considered to be the source of the carbon incorporated in the alloy coatings. However, as mentioned above, glycine did not directly participate in the cathode reactions. Thus, it is speculated that glycine may have undergone other reactions and been involved in the electrodeposition process in the form of new compounds. Marangoni et al. [30] and Hasegawa et al. [31] reported that free glycine molecules in the electrolyte will undergo side reactions on the surfaces of Pt anodes, resulting in oxidative decomposition. In this process, glycine decomposes through the Kolbe reaction followed by hydrolysis to form formaldehyde during the first step of oxidation; during this process, ammonium is formed along with carbon dioxide. Moreover, formaldehyde is possibly further oxidized at the anode to form formic acid [30]. Thus, the oxidation reactions of glycine at the anode were considered to be the following:

CH_2_(NH_3_^+^)COOH + H_2_O → CH_2_O + 2H^+^ + NH_4_^+^ + CO_2_(gas)↑ + 2e^−^, (2)

CH_2_O + H_2_O → HCOOH + 2H^+^ + 2e^−^. (3)

**Figure 7:**
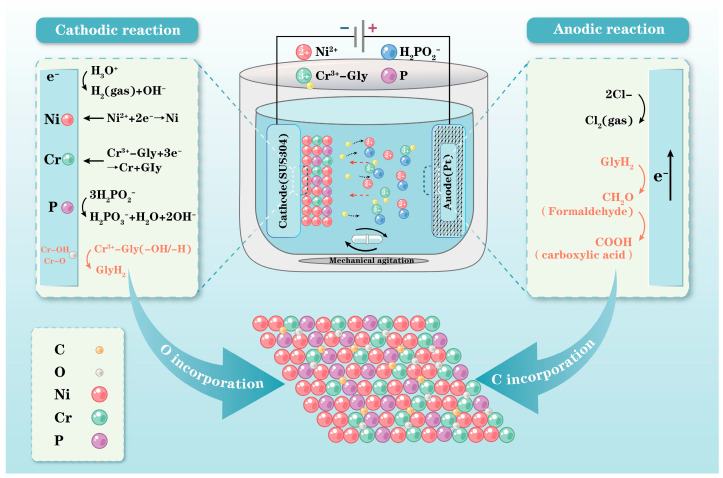
Electrodeposition mechanism of Ni-Cr-P alloys: (**top**) cathodic/anodic reactions and (**bottom**) illustration of incorporation behavior of impurities.

Consequently, it is speculated that formaldehyde and formic acid were possible sources of carbon inclusions. Actually, formic acid and formaldehyde have been employed as the source of carbon for Cr-based alloy electrodeposition [19,32]. Moreover, Kumar et al. [32] confirmed the bond formation between formaldehyde and metallic chromium by Fourier-transform infrared spectroscopy studies. Elemental analyses of the Ni-Cr-P and Ni-P alloys electrodeposited from electrolytes with and without a Cr source were performed. A non-negligible amount of carbon was detected in the Ni-Cr-P alloy, but no carbon was detected in the Ni-P alloy (as shown in Table 4). Therefore, it can be speculated that the incorporation behavior of carbon was closely related to trivalent chromium electrodeposition. It should be noted that the Cu detected from the Ni-P and Ni-Cr-P alloys originated from the Cu substrate. Furthermore, the small amount of O was unlikely to have come from the metal oxides contained in the Ni-P alloy but most likely came from the oxides on the Cu surface. In addition, since the carbonyl groups contained oxygen, the carbonyl groups included in the Ni-Cr-P interlayers seemed to be a minor source of oxygen. Based on the main reactions on the surfaces of the electrodes and the incorporation behavior of impurities, the electrodeposition mechanism of Ni-Cr-P alloys is illustrated in Figure 7.

### 3.2. Formation of Brazed Joint

#### 3.2.1. Effect of Impurities on Brazed Joint

**Figure 8:**
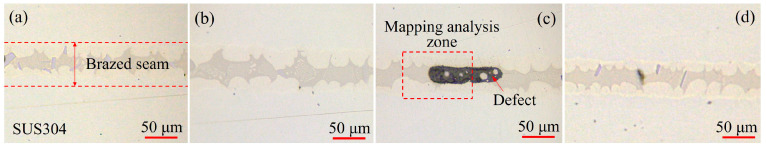
Optical microscopy (OM) images of the cross-section of the joint brazed without preheating the brazing sheets: (**a**) left side of the brazed joint, (**b**,**c**) center of the brazed joint, and (**d**) right side of the brazed joint.

**Figure 9:**
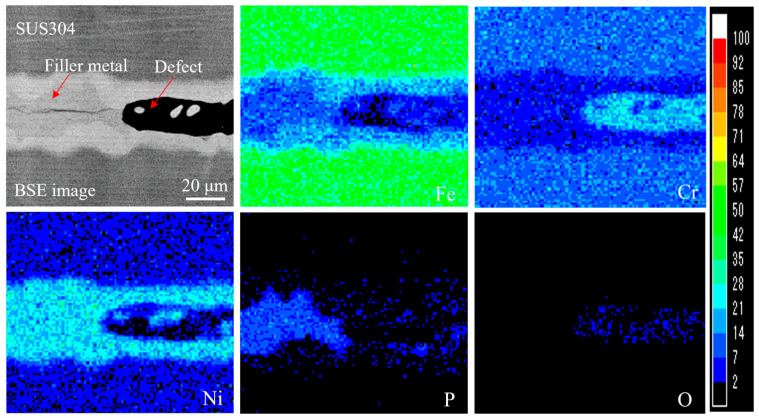
SEM image and corresponding elemental distributions of the cross-section of the SUS304 joint brazed without preheating the brazing sheets (corresponding to the observation zone in Figure 8c).

Figure 8 shows the OM images of the cross-section of an SUS304 joint brazed without preheating the brazing sheets. It was found that tiny defects were formed throughout the brazed seam, as shown in Figure 8c. To understand the reasons for the formation of these defects, an elemental mapping analysis was performed, and the results are shown in Figure 9. There was almost no Fe, Ni, or P present, but large amounts of Cr and O filled in the blanks of the seam. Subsequently, an XRD analysis of the fractured surface of the brazed joint was performed to obtain a deeper understanding of this phenomenon.

**Figure 10:**
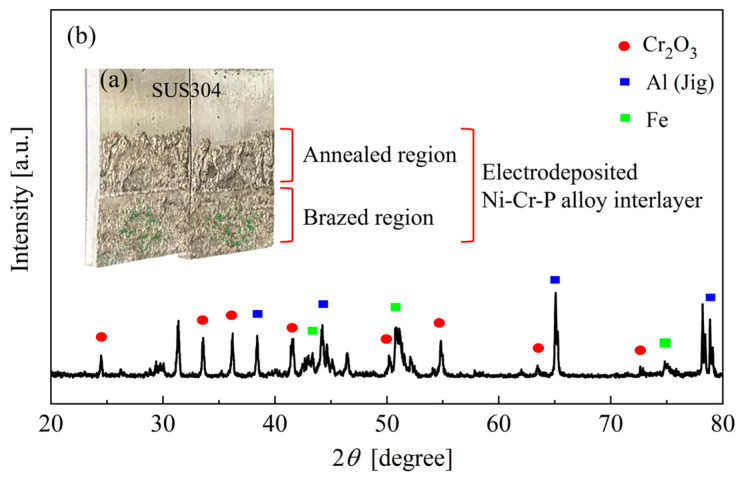
Brazing characteristic analysis of the electrodeposited Ni-Cr-P interlayer: (**a**) macrograph of a fractured surface of the joint brazed without preheating the brazing sheets and (**b**) X-ray diffraction (XRD) pattern of the brazed region in the fractured surface.

To analyze the brazing characteristics of the electrodeposited Ni-Cr-P interlayer, a portion of the alloy interlayer was exposed to a H_2_ atmosphere. Figure 10a shows the macrograph of a fractured surface of the brazed joint. It was found that a small amount of green powder was scattered on the brazed region. In contrast, the Ni-Cr-P interlayer exposed to the H_2_ atmosphere (i.e., the annealed region) did not form any green powder but showed a bright metallic luster. This means that H_2_ gas could effectively inhibit the formation of the green powder. The physical phase analysis results of the green powder are shown in Figure 10b. The weaker peaks in the XRD spectra at 24.5°, 33.6°, 36.4°, etc. (marked as red dots in Figure 10b), suggested that the green powder was Cr_2_O_3_. Cr_2_O_3_ has a high melting point of approximately 2435 °C and did not melt at the brazing temperature of 1020 °C. The generated Cr_2_O_3_ powder agglomerated in the brazing seam, blocking the flow of liquid filler and forming the defects shown in Figure 8.

From the above analysis of the joint, it can be inferred that some chemical reactions may have occurred in the seam under the catalytic effect at high temperatures during brazing. These harmful reactions seemed to be related to the oxygen incorporated in the electrodeposited Ni-Cr-P interlayer. As discussed in Section 3.1.3, the incorporated O exists mainly in the form of chromium hydroxide in the interlayer. Chromium hydroxide was easily decomposed by heat and could be completely decomposed into Cr_2_O_3_ at 400–500 °C. Therefore, the decomposition of chromium hydroxide during brazing was most likely an important source of Cr_2_O_3_:

2Cr(OH)_3_ → Cr_2_O_3_ + 3H_2_O(gas)↑. (4)

On the other hand, the EPMA mapping analysis and XRD results did not show any evidence of the presence of carbon in the brazed joint. Combined with the discussion of the carbon incorporation behavior in Section 3.1.3, this finding strengthens the notion that carbonyl compounds were directly incorporated into the electrodeposited interlayer in a molecule pattern and were linked to metallic chromium. In the brazing process, the carbon precipitates seemed to be eliminated from the alloy interlayer via outgassing due to the heat treatment because of the weak bonds between the carbonyl groups and chromium. Consequently, determining how to eliminate the oxygen in the electrodeposited Ni-Cr-P interlayer is worth more attention.

#### 3.2.2. Effect of Preheating on Brazed Joint

**Figure 11:**
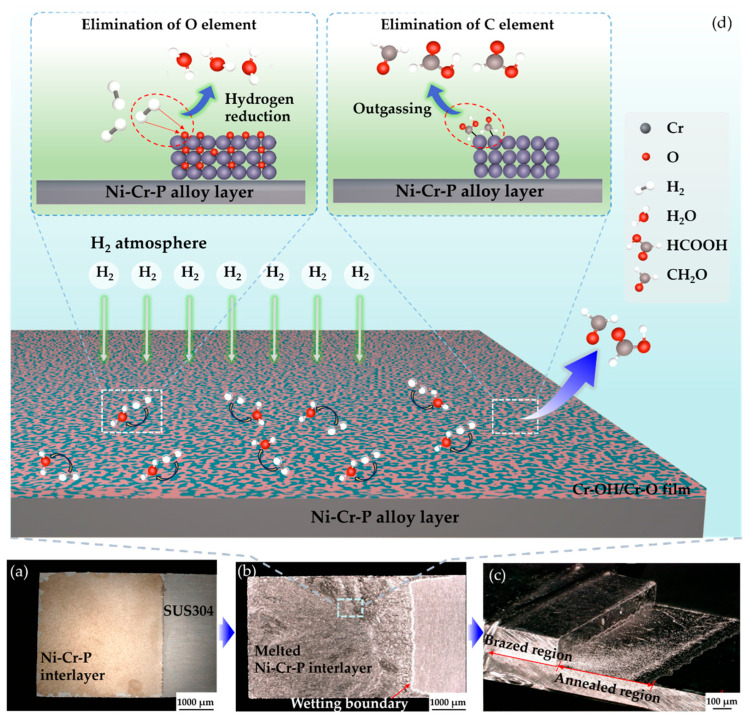
Process flow of step-wise brazing method: (**a**) preparation of brazing sheet by electroplating, (**b**) annealing of brazing sheet (i.e., preheating), (**c**) actual brazing, and (**d**) schematic diagram of elimination of impurities during the annealing process.

To eliminate the effect of impurities in the electrodeposited interlayer on the brazed joint, preheating was performed on the brazing sheets before actual brazing. Figure 11a–c show images of the materials at different steps of the brazing process in the step-wise method. First, a brazing sheet was prepared by electrodepositing a Ni-Cr-P interlayer onto one surface of SUS304. The alloy interlayer was subsequently annealed under brazing temperature, during which the strong reducing properties of H_2_ gas were utilized to remove oxygen from the alloy interlayer, thereby avoiding the formation of green Cr_2_O_3_ powder. The reaction mechanism involved in this process was as follows:

Cr_2_O_3_ +3H_2_→ 2Cr + 3H_2_O(gas)↑. (5)

Combined with the carbon precipitate removal mechanism discussed in Section 3.2.1, the elimination mechanism of impurities during annealing of the electrodeposited Ni-Cr-P interlayer in a hydrogen atmosphere is shown in Figure 11d. In summary, when the alloy interlayer is heated from room temperature to annealing temperature, chromium hydroxides (i.e., O impurities) formed during the electrodeposition stage will decompose into stable chromium oxide (i.e., Cr_2_O_3_); subsequently, during the annealing stage, Cr_2_O_3_ will be reduced to metallic chromium atoms by hydrogen under the catalytic action of high temperature. The reduced metallic chromium will continue to participate in the joint formation. Simultaneously, the carbonyl compounds (i.e., C impurities) weakly bonded with metallic chromium will be discharged from the molten interlayer in the form of outgassing.

In addition, as shown in Figure 11b, the melted alloy interlayer spread evenly on the steel surface, showing a clear wetting boundary without any local shrinkage, indicating that the electrodeposited Ni-Cr-P interlayer had good wettability on the SUS304 surface. Finally, the as-annealed brazing sheets were assembled for brazing to form a joint with a good appearance (see Figure 11c).

**Figure 12:**
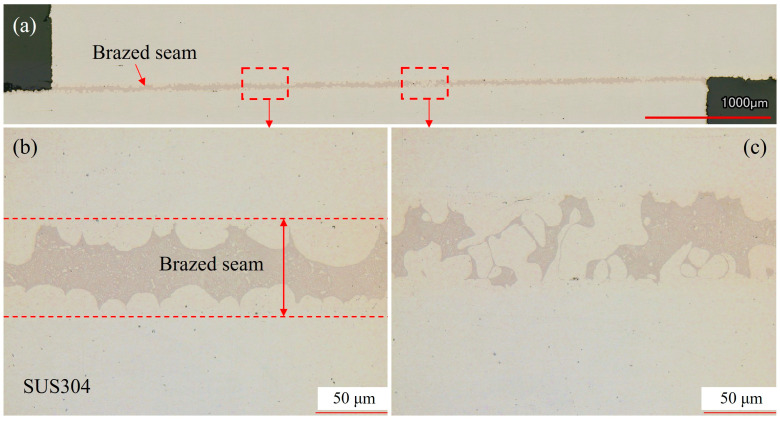
OM images of the cross-section of the brazed joint obtained by the step-wise method: (**a**) overview of the brazed joint and (**b**,**c**) magnified zones of the brazed joint.

Figure 12 shows the OM images of the cross-section of the joint brazed with the step-wise method. Based on the overall view of the brazed joint shown in Figure 12a, the filler metal showed good bondability without any defects forming in the seam, indicating that the brazing of SUS304 with the electrodeposited Ni-Cr-P interlayer was successfully realized. The magnified regions (see Figure 12b,c) show more detailed information about the brazed seam, demonstrating the reliability of the step-wise brazing process.

### 3.3. Microstructure Characterization

**Figure 13:**
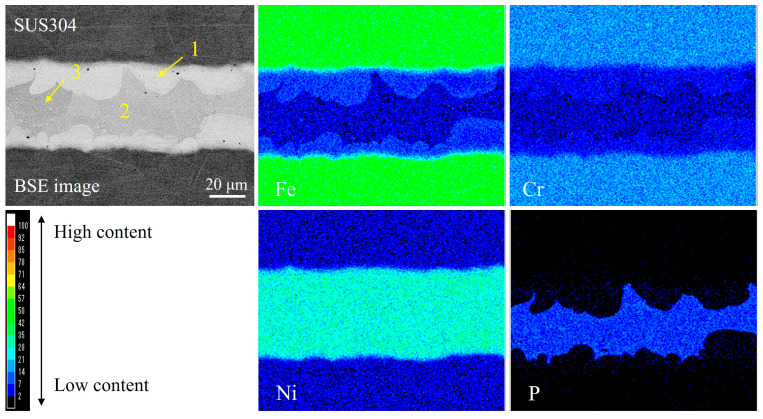
Elemental distributions of the cross-section of the joint brazed with the step-wise method.

**Figure 14:**
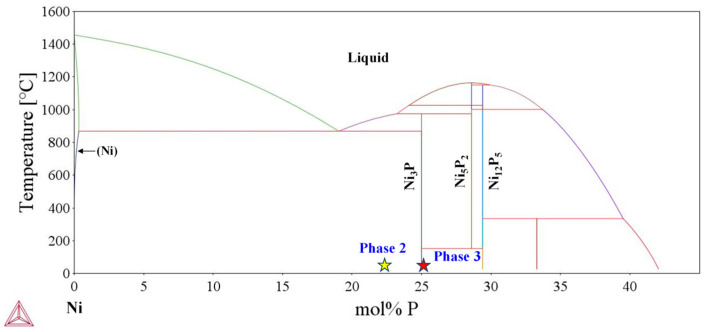
Phase diagram of binary Ni-P alloys calculated by Thermo-Calc 2021 (in 25 °C); the stars in the phase diagram indicate the compositions of these phases.

Figure 13 shows the elemental mapping analysis of the cross-section of the brazed joint. From the backscattered electron (BSE) image, it seems that there were mainly three phases that formed in the brazed seam. One was the bright gray phase (marked as 1), which was formed on the interfaces of SUS304 and mainly contained Ni, Fe, and Cr. The others were the light gray and gray phases (marked as 2 and 3, respectively) distributed in the filler metal region, mainly containing Ni and P. The chemical compositions of each phase are given in Table 5. Phase 1 had a composition ratio of Ni/Fe/Cr/P = 82.05:8.98:7.47:1.51. Since Ni and Fe had unlimited solubility with each other, while Cr had limited solubility with Ni and Fe, the phase was identified as a Ni–Fe-Cr solid solution [33]. Phase 2 had a composition ratio of Ni/Fe/Cr/P = 69.64:3.48:4.21:22.68, and the Ni/P ratio was approximately 3. According to the calculated Ni-P binary phase diagram, shown in Figure 14, the phase can be identified as the Ni_3_P eutectic phase. In addition, there were a large number of white microparticles distributed in phase 2 with the same composition as the bright gray phase 1 (see the BSE image in Figure 13), making the gray phase 3 seem slightly different from phase 2. Accordingly, this led to a slight increase in the contents of Ni and Fe and a slight decrease in the content of P (shown in Table 5). The Ni/P ratio of phase 3 was approximately 2.5. From the Ni-P phase diagram, it was inferred that phase 3 was a Ni_3_P eutectic phase containing a small quantity of Ni_5_P_2_.

### 3.4. Strength Property of Brazed Joint

#### 3.4.1. Shear Strength

Shear tests were performed to evaluate the mechanical properties of the joint brazed with the step-wise method. Three specimens were prepared for the shear tests, and the average strength of the joints was determined to be 63 MPa. The standard deviation of the shear strength was 2.14 MPa, indicating that the shear strength of the brazed joint with the electrodeposited Ni-Cr-P alloy interlayer is relatively stable. The shear strength was lower than that of other reported joints brazed with Ni-Cr-P filler metal, as listed in Table 6. As is well known, the shear strength of a brazed joint depends on many conditions, such as the brazing temperature, holding time, and gap width (or the filler metal thickness). Zhang et al. [2] and Wu et al. [34] reported that the shear strength of a brazed joint increases with increasing brazing temperature. This can be associated with the fact that increasing the brazing temperature promotes the diffusion of elements between the filler metal and the base material, promotes the formation of phases with better mechanical properties, and improves the strength properties of the joints. Similarly, utilizing different brazing times or gap widths can also contribute to improving the shear strengths of the joints by modifying the microstructural properties in the brazed seams. However, from a performance perspective, the brazed joints prepared in this work were of good quality without any defects, which provides meaningful guidance for practical applications. The optimization of the brazing conditions will be studied in detail in our future work according to application requirements.

#### 3.4.2. Fracture Mode

**Figure 15:**
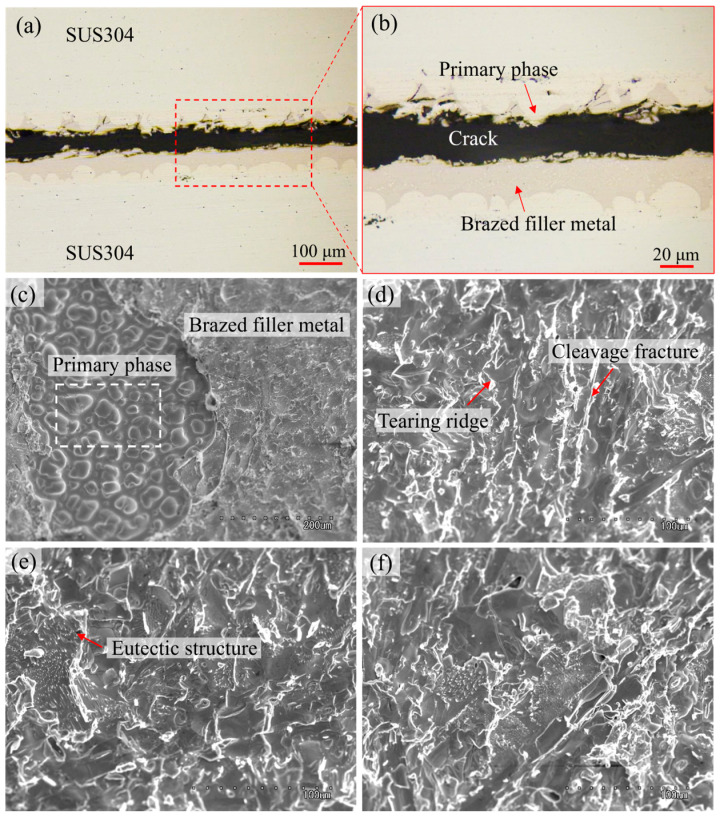
Fracture mode observation of SUS304 joints brazed with electrodeposited Ni-6Cr-11.1P interlayers: (**a**,**b**) OM images of the cross-section of the fractured joint and (**c**–**f**) SEM images of the fracture surface.

The fracture mode observations of SUS304 joints brazed with electrodeposited Ni-6Cr-11.1P interlayers are shown in Figure 15. As shown in Figure 15a,b, cracks were generated at the interface between the brittle compounds (P-rich phases) and the ductile primary phase (Ni-Fe-Cr solid solution). The Ni-Fe-Cr solid solution, with a strong plastic deformation ability, could alleviate the stress concentration of the brazed joint and form many curved tearing edges, hindering the direct propagation of cracks in the brittle phase and the encountering of local cracks. This was beneficial for increasing the joint strength. The fractured surface was characterized by large areas of the primary phase exposed on the surface (Figure 15c), corresponding to the above-mentioned crack extension characteristics. When the crack propagated to the P-containing intermetallic compounds, the hard and brittle characteristics of these compounds made it easy for cleavage fractures to occur (see Figure 15d), eventually leading to breakage. However, a eutectic structure could be observed on the fracture surface (see Figure 15e,f), and almost no unmelted filler metal was found on the fractured surfaces, indicating that the electrodeposited Ni-Cr-P interlayers were completely melted during brazing. The existence of cleavage facets and tearing ridges on the fracture surface corresponded to quasi-cleavage fracture characteristics.

## 4. Conclusions

The incorporation mechanism of impurities (i.e., oxygen and carbon) during the electrodeposition of Ni-Cr-P alloys from a glycine–Cr(III) solution was studied. The oxygen incorporated into the electrodeposited Ni-Cr-P alloys mainly came from the Cr(III)–hydroxy complexes that were formed by the hydrolysis and olation between the Cr(III) complexes and OH^−^ near the cathode. Glycine decomposed into formaldehyde and formic acid on the anode surface. These carbonyl compounds were directly incorporated into the electrodeposited alloy interlayers in a molecule pattern and formed weak links with the metallic chromium, which became a pathway for carbon incorporation.

A small amount of green powder, whose physical phase was confirmed to be Cr_2_O_3_, formed in the brazed seam when the brazing sheets were not preheated. These compounds were formed by the decomposition of chromium hydroxides in the alloy interlayers under high-temperature catalysis. In addition, through the analysis of the brazing characteristics of the electrodeposited Ni-Cr-P alloy interlayers, it was found that the strong reducing property of hydrogen could effectively inhibit the formation of Cr_2_O_3_.

The brazing of thin-walled SUS304 sheets using electrodeposited Ni-Cr-P alloy interlayers as the filler metal was successfully realized by a step-wise method. To eliminate the impurities in the electrodeposited alloy interlayers, the brazing sheets were annealed to remove the oxygen in the interlayers by utilizing the strong reducing effect of hydrogen. Simultaneously, the weak links between the carbonyl groups and the chromium atoms were broken under the catalysis effect at high temperatures, and carbon was discharged from the interlayers via outgassing.

An excellent joint with a shear strength of 63.0 MPa was obtained. The brazed seam was mainly composed of a Ni-Fe-Cr solid solution at the brazed interfaces of the SUS304 sheets. The Ni_3_P eutectic phase formed in the brazed filler zone, and small amounts of the Ni_5_P_2_ phase were scattered in the Ni_3_P eutectic phase. Fracture mode analysis showed that the cracks extended along the interface between the brittle P-containing phases and the primary phase, resulting in fracture. The existence of cleavage facets and tearing ridges on the fracture surface corresponded to quasi-cleavage fracture characteristics.

## Figures and Tables

**Table 1:** Bath composition and experimental conditions for the electrodeposition of the Ni-Cr-P alloy.

Composition	Concentration (M)				Electrodeposition Conditions
	S1	S2	S3	S4	
CrCl_3_·6H_2_O	0.4	0.35	0.4	0.5	Current density: 10, 15, 20 A/dm^2^ pH: 1.8 Bath temperature: 30 °C Plating time: 70–150 min Anode: Pt mesh Cathode: SUS304/Cu Electrode distance: 2.5 cm
NiCl_2_·6H_2_O	0.25	0.3	0.25	0.126	
NaH_2_PO_2_·H_2_O	0.14	0.14	0.14	0.24	
C_2_H_5_NO_2_ (Glycine)	1.0	0.5	0.5	0.5	
C_6_H_5_Na_3_O_7_	0.2	0.2	0.2	-	
NH_4_Cl	0.5	0.5	0.5	0.5	
NaBr	0.145	0.145	0.145	0.145	
NaCl	0.5	0.5	0.5	0.5	
H_3_BO_3_	0.5	0.5	0.5	0.5	
C_7_H_4_NO_3_SNa·2H_2_O	0.6 g/L	0.6 g/L	0.6 g/L	0.6 g/L	
SDS	0.1 g/L	0.1 g/L	0.1 g/L	0.1 g/L	

**Table 2:** Test solutions used to perform ultraviolet–visible (UV-vis) spectrometry.

Composition	Concentration (M)	
	Cr	Cr + Glycine
CrCl_3_·6H_2_O	0.4	0.4
NiCl_2_·6H_2_O	-	-
C_2_H_5_NO_2_ (Glycine)	-	0.5
NH_4_Cl	0.5	0.5
NaBr	0.145	0.145
NaCl	0.5	0.5
H_3_BO_3_	0.5	0.5
C_7_H_4_NO_3_SNa·2H_2_O	0.6 g/L	0.6 g/L
SDS	0.1 g/L	0.1 g/L

**Table 3:** Chemical compositions of SUS304 and electrodeposited Ni-Cr-P alloy interlayer (mass%).

Symbol	Ni	Cr	P	S	Si	Mn	C	O	Fe
SUS304	8.00–10.50	18.00–20.00	≤0.045	≤0.03	≤1.00	≤2.00	≤0.08	-	Bal.
Ni-Cr-P	Bal.	6.00	11.10	-	-	-	2.80	2.70	-

**Table 4:** Chemical compositions of Ni-Cr-P and Ni-P alloys electrodeposited from electrolytes (S3 in Table 1) with and without a Cr source.

	Ni (Mass%)	Cr (Mass%)	P (Mass%)	O (Mass%)	C (Mass%)	Cu (Mass%)
Ni-P	81.9	-	12.2	1.2	-	4.7
Ni-Cr-P	21.4	15.1	7.6	14.7	2.2	39

**Table 5:** Chemical composition of the analyzed points in the SUS304 brazed joint.

Points	Element Composition (mol%)				Possible Phase
	Ni	Cr	P	Fe	
1	82.05	7.47	1.51	8.98	Ni-Fe-Cr solid solution
2	69.64	4.21	22.68	3.48	Ni_3_P eutectic phase
3	68.16	3.42	25.35	3.07	(Ni_3_P + little Ni_5_P_2_) eutectic phase

**Table 6:** Comparison of the shear strengths of brazed joints with different brazing conditions.

Ref.	Base Metal	Interlayer Thickness (μm)	Temperature (°C)	Time (min)	Shear Strength (MPa)
This work	SUS304	Ni-Cr-P (20)	1020	20	63
[2]	HNS	Ni-Cr-P (50)	950, 1000, 1050	20	60–163
[3]	SUS304	Ni-Cr-P + 3%Cu (30, 100)	940, 960, 980	5, 30	84–119
[34]	Super-Ni/NiCr laminated composite/SUS304	Ni-Cr-P (-)	940, 980, 1040	20	36–137
[35]	SUS304/Mo-Cu composite	Ni-Cr-P (30)	980	20	155

Note: the total time for step-wise brazing = annealing (10 min) + brazing (10 min).

## Data Availability

The original contributions presented in this study are included in the article. Further inquiries can be directed to the corresponding author.
